# Enhanced distance protection for HVDC lines using adaptive neuro-fuzzy inference systems

**DOI:** 10.1371/journal.pone.0338629

**Published:** 2026-01-05

**Authors:** A. M. Hamada, M.I. Abdel-fattah, Ali M. El-Rifaie, Fahmi Elsayed, Mohsen A. M. El-bendary, Tamer A. A. Ismail, Ijaz Ahmed, M. M. R. Ahmed

**Affiliations:** 1 Railway Department, Faculty of Industrial and Energy Technology, Borg El-Arab Technological University, Alexandria, Egypt; 2 Electrical Technology Department, Faculty of Technology and Education, Helwan University, Helwan, Egypt; 3 Electrical Engineering Department, College of Engineering and Technology, American University of the Middle East, Egaila, Kuwait; 4 Electrictronics Technology Department, Faculty of Technology and Education, Helwan University, Helwan, Egypt; 5 Electrical Department, Faculty of Technology at El Sahafa Street, Ministry of Higher Education, Cairo, Egypt; 6 Interdisciplinary Research Center for Sustainable Energy Systems, King Fahd University of Petroleum and Minerals, Dhahran, Saudi Arabia; University of Bonab, IRAN, ISLAMIC REPUBLIC OF

## Abstract

Accuracy and speed of fault detection are crucial to the performance of DC transmission systems. In this paper, a novel approach is proposed for fault detection, classification, and localization in high-voltage DC transmission lines (HVDC-TLs). The proposed approach for protecting HVDC-TLs by designing and operating a distance protection scheme has been constructed using a fuzzy inference system and training an adaptive neuro-fuzzy inference system. A fuzzy inference system model is proposed to detect faults, classify them, and determine the zone where the fault occurred. The transmission line is divided into three zones to facilitate fault location identification. An adaptive neuro-fuzzy inference system is then trained to determine the fault location per kilometer. The proposed distance protection scheme identifies faults with high fault resistance; it can be successfully used to estimate the fault area and locate faults in HVDC-TLs using the concept of fuzzy inference. A monopolar DC transmission line system was modeled and operated, and several faults were simulated using PSCAD and MATLAB software. As clarified from the various simulation experiments, the proposed approach has performed better than the existing techniques and recently published related works.

## 1. Introduction

**Fig 1:**
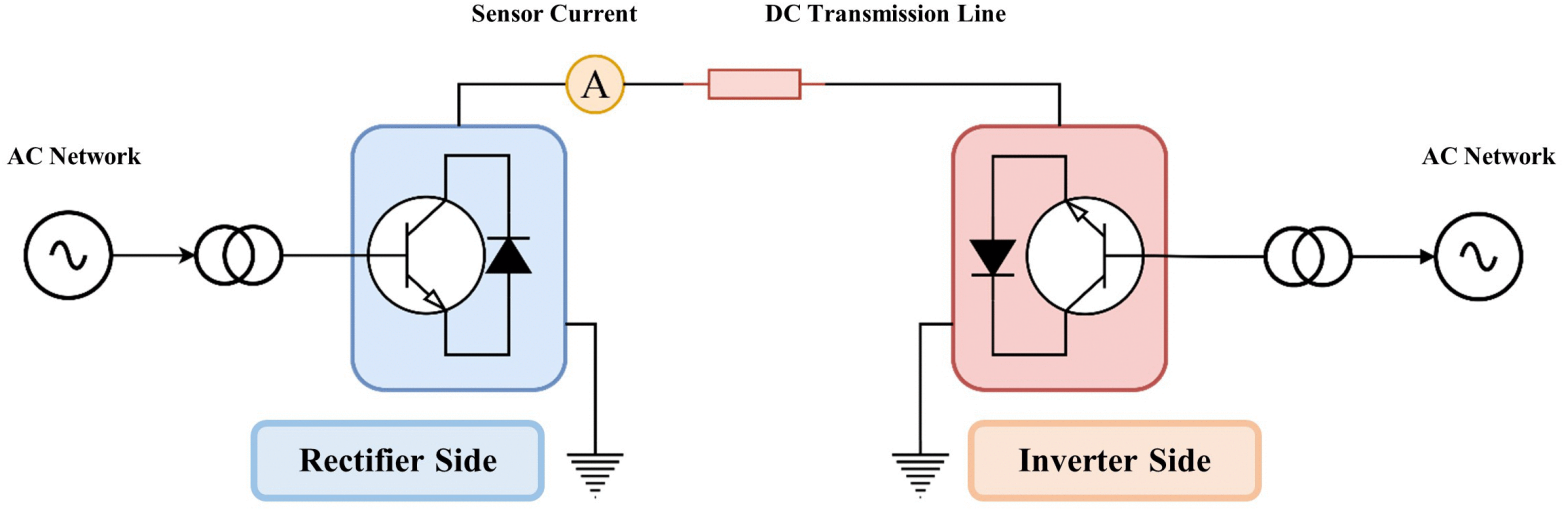
Monopolar DC transmission line contents.

HVDC transmission systems are increasingly recognized as a key solution for long-distance and high-capacity power transfer. Compared with conventional high-voltage alternating current (HVAC) transmission, HVDC offers a number of technical and economic advantages. It reduces transmission losses, requires fewer conductors, and eliminates problems related to phase angle, synchronization, and transient stability [1]. Therefore, HVDC technology is widely adopted in various applications such as interconnecting asynchronous power grids, transmitting electricity from remote renewable energy sources, and enabling efficient bulk power transfer over long distances. A typical HVDC link employs converter stations at both ends of the transmission line, as illustrated in Fig 1. At the sending end, rectifiers convert AC power into DC, which is transmitted over the line; at the receiving end, inverters convert the DC back into AC. This bidirectional flow capability makes HVDC systems both flexible and efficient in the modern power networks [2].

Despite these advantages, HVDC transmission lines remain vulnerable to faults from lightning, equipment failure, insulation breakdown, or human error. Such events can disrupt power delivery, damage equipment, and cause major financial losses. Accurate fault detection, classification, and location are critical to ensure reliability, enable rapid isolation and restoration; therefore, inefficient protection may lead to prolonged outages and reduced system stability [3,4]. Table 1 highlights the symbolic representation of technical terms utilized in the manuscript.

**Table 1:** Description of symbols and abbreviations.

Symbol	Description
HVDC-TL	High voltage direct current transmission lines
FIS	Fuzzy inference system
ANFIS	Adaptive neuro fuzzy inference system
I_ins_	Instantaneous value
I_rms_	Root mean squared value
SNR	Signal-to-noise ratio
TS	True status
FS	False status
SCR	Short Circuit Ratio
gbellmf	Generalized bell membership function
trimf	Triangle membership function

Over the past decades, a variety of fault detection and location strategies have been proposed for HVDC systems. These methods can be broadly categorized into parameter-based, frequency component based, travelling wave-based, and knowledge-based approaches [5,6]. The parameter-based approaches, such as virtual impedance-based methods [7] and time-domain impedance-based methods [8], often yield large estimation errors because they heavily depend on network parameters. To overcome these limitations, frequency component-based methods like gap frequency spectrum analysis (GAP) [9] and travelling wave (TW)-based techniques have been introduced [10–13]. TW methods are among the most widely used in HVDC systems due to their speed and accuracy [14,15], but their performance is influenced by wave-front arrival times, monitoring device placement, fault resistance, and line characteristics [16–20].

Recently, knowledge-based intelligent models, particularly the ANFIS, have been applied to address these issues. ANFIS techniques offer high accuracy and fast response because they are trained on system data and remain effective under varying fault conditions [21]. They also enable accurate fault classification when trained on diverse fault scenarios [22]. However, ANFIS performance depends on the training strategy and the amount of fault data available [23]. These limitations can be mitigated by using heuristic and meta-heuristic optimization methods for training [24,25] and employing feature extraction techniques to enhance fault signal representation [26].

Several feature extraction methods have been proposed in previous studies [27]. In VSC-HVDC systems, hybrid schemes combining ANFIS with optimal parameter selection and Hilbert–Huang (HH) transformations have been proposed for fault location [28]. A non-unit DC distance protection technique has also been introduced, estimating fault distance by analyzing DC voltage transient frequency using HHT. Simulation results demonstrated that this method achieved a correct operating rate of > 94% for faults within the second protection zone [29]. Furthermore, the wavelet transform (WT) has been widely used for extracting fault signal features in different applications [30,31].

Based on the previous, our research paper presents a new fault detection, classification, and location approach with robustness and lower complexity features. This proposed approach directly utilizes DC current signals and remains accurate under fault resistances up to 300 Ω and noisy environments. It is structured in two stages: first, a fuzzy inference system (FIS) divides the HVDC transmission line into three protection zones and identifies the fault zone; second, ANFIS is trained to determine the precise fault location along the line.

The remainder of the paper is organized as follows: Section 2 introduces the ANFIS framework, Section 3 describes the VSC-HVDC model and implementation steps, Section 4 presents the ANFIS-based fault location design, Section 5 validates performance through PSCAD and MATLAB simulations under various operating conditions, and Section 6 concludes with key findings.

## 2. Adaptive neuro-fuzzy inference system (ANFIS)

In this section, a historical note about ANFIS has been presented and with its main contents. Jang introduced and developed ANFIS, a fuzzy inference system, within the paradigm of adaptive networks in 1993. ANFIS makes use of ANN learning power to extract fuzzy if-then rules with appropriate membership functions (MF) from training input-output pairs, resulting in inference. In general, ANFIS is divided into two sections: the preceding section and the epilogue section. Within the ANFIS network topology, ambiguous rules bind the preceding and outcome segments together. The parameters in these areas are used to train and update ANFIS. More details about ANFIS can be found at [32].

**Fig 2:**
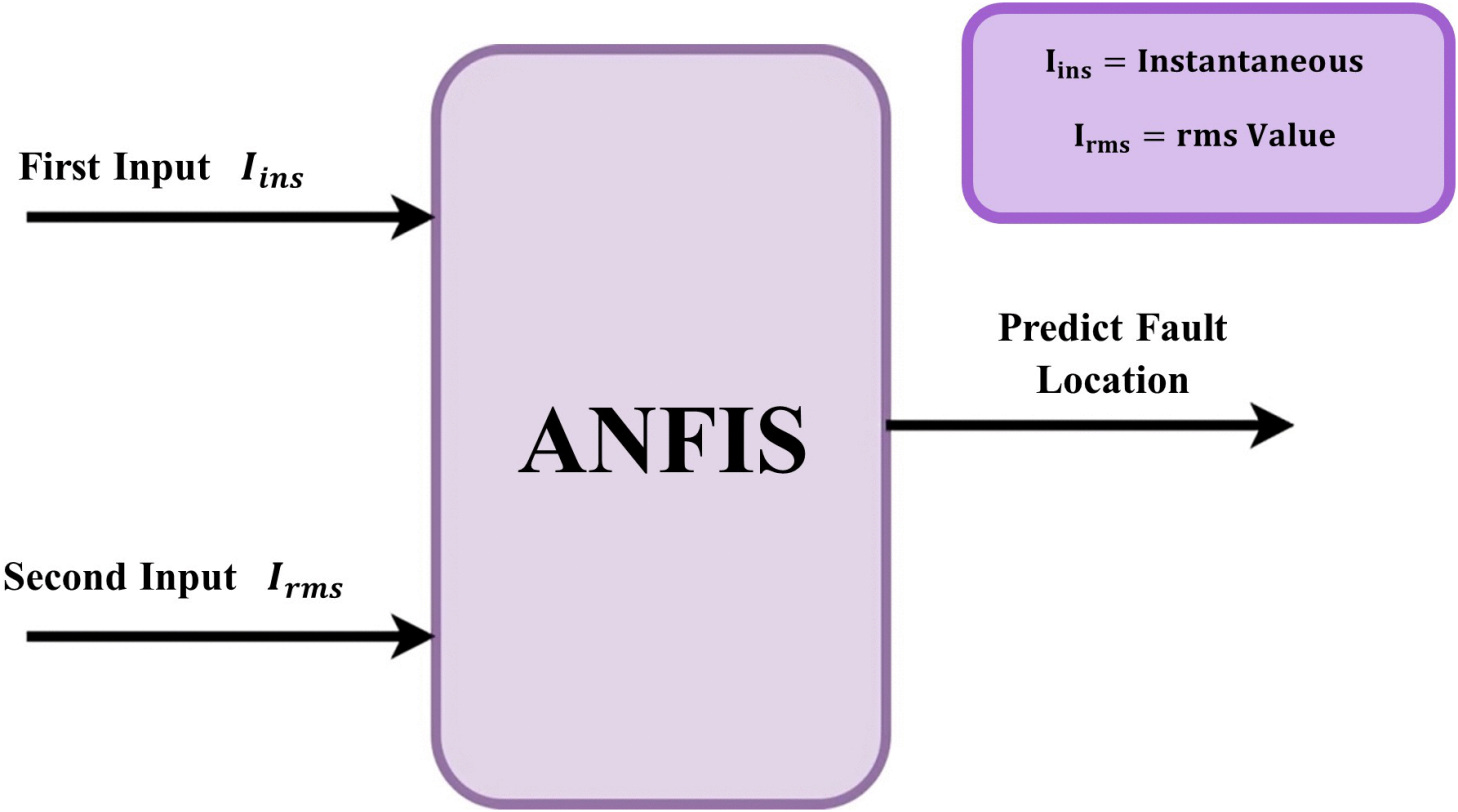
The structure of the input and output of ANFIS in the proposed principle.

Fig 2 shows the input and output of ANFIS in the proposed principle, where *I*_*ins*_ is the first input of the current signal (Instantaneous value), *I*_*rms*_ The second input of the current signal (rms value), and the output is the predicted fault location. It was decided to use the input current signals I_rms_ and I_inst_ since they are the most physically relevant values that undergo significant change under various fault scenarios. These inputs enable the ANFIS model to precisely identify different fault types and pinpoint their locations by capturing the system’s behavior in both steady and unstable states.

## 3. The proposed approach

The efficient proposed fault detection/location has been discussed in this section based on the ANFIS system.

**Fig 3:**
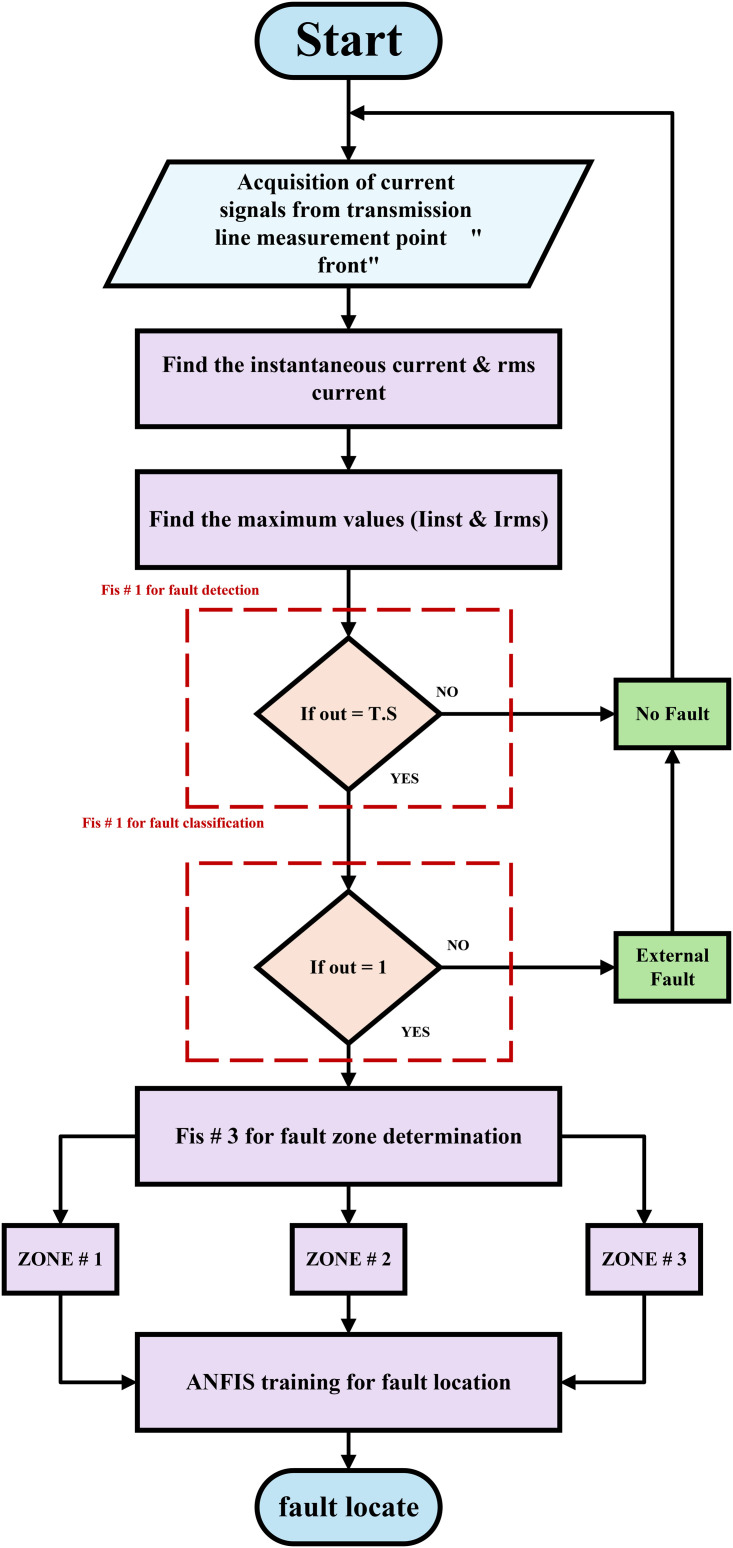
Flowchart of the proposed fuzzy-based method.

**Fig 4:**
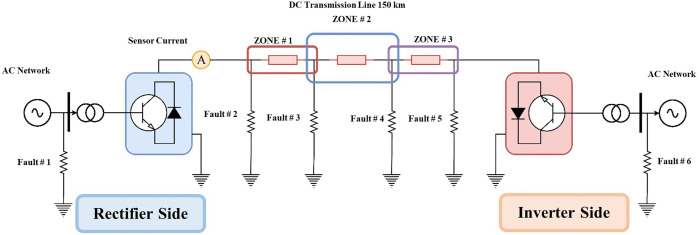
Monopolar structure system for the proposed method.

To detect and locate the fault, the suggested method adopts a fuzzy inference system. A flowchart of the suggested fuzzy-based method is shown in Fig 3. The current signals are acquired from the transmission line in the manner described in Fig 4. The signals obtained are sent into fuzzy inference systems (FIS) and adaptive neuro-fuzzy inference systems (ANFIS) as inputs. Table 2 shows the HVDC TL parameters

**Table 2:** The HVDC-TL parameters and constants.

Parameter	Value
The type of study system	Monopolar system
The type of converter	Twelve pulses
Reactive power support	Fixed capacitors
AC filters	Damper filter
SCR (Control Ratio)	Rectifier: 2.5 & Inverter:2.5
DC voltage system	500 kV
DC current	2 kA
Rated power	1000 MW
Transmission line length	150 km
Resistance per km	0.03206 ohm/km

**Fig 5:**
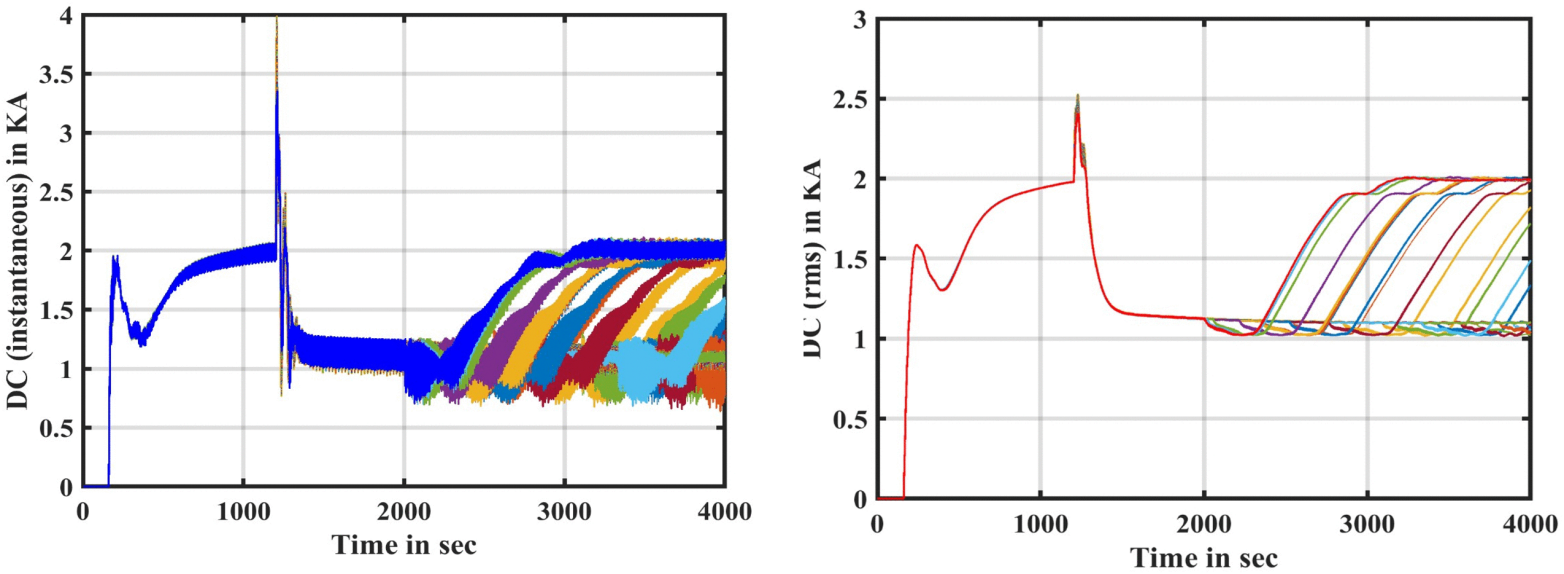
Total DC signal at faults on the transmission line simultaneously: Instantaneous current in the HVDC-TL, and RMS value of the current in the HVDC-TL.

Several faults were applied along the transmission line, with the time of the fault being fixed at 0.3 sec and the prolongation of the fault continuation time to 0.2 sec. During the period of the fault, the maximum value of the instantaneous current and the maximum effective value of it are obtained as shown in Fig 5. When a DC transmission system has a failure, the voltage value falls while the current value rises; this characteristic can be considered advantageous in the fault detection of HVDC-TL systems. The proposed fault detection/location approach has been evaluated under the various system’s operating conditions, such as a variation in fault AC/DC types, fault location, fault resistance, far-end faults, and near-end faults, as clarified in the simulation experiments section. It also necessitates the use of three fuzzy inference system controllers to enhance the accuracy of the proposed approach. In addition, in the training stage of the adaptive neural fuzzy inference system for fault location, three separate tasks have been determined: fault detection, fault section identification, and establishing the fault location were performed. Because each of these relay jobs is Due to the three different tasks, three independent fuzzy controllers (FIS-1, FIS-2, and FIS-3) are utilized in our proposed approach, each one of them responsible for a distinct task. The FIS-1 is designed to detect faults in both the DC and AC sides, the FIS-2 is designed to identify fault sections [33], and the FIS-3 is designed to find the fault zone by training the ANFIS. If there is an AC or DC fault section, the fault detection output is ‘1’. Because there is no fault, the fault detection output is “0.”

Each fuzzy unit contains nine rules (3 × 3), with each unit using two inputs and three trigonometric membership functions (LOW, MDT, and HIGH) per input. For the FIS-1 and FIS-2 fault detection and classification modules, the zero-order Sugeno output membership functions are constant values corresponding to the fault flags (–1, 0, 1), where 0 indicates no fault, 1 indicates an internal fault, and –1 indicates an external fault. For fault zone selection, the second input and single output correspond to zone identifiers (1, 2, 3) representing Zone 1 (output ≃ 1), Zone 2 (output ≃ 2), and Zone 3 (output ≃ 3). The number and structure of fuzzy rules, as well as the membership functions and their parameterization, are clearly presented in Table 3. In the following sub-section, the various models of FIS-n have been described separately.

**Table 3:** Summary of FIS structure, rule base, and membership function parameters.

Item		The details of training	
		Description	Setting
Fault detection & classification	Input data	Two inputs	[LOW, MDT, HIGH]
	Output data	Single output	[External fault, No fault, Internal fault]
Zone faulty	Input data	Two inputs	[LOW, MDT, HIGH]
	Output data	Single output	[Zone # 1, Zone # 2, Zone # 3]
Type of membership function		For input data	Triangle membership function ‘trimf’
Number of membership functions		Membership function/input	3 & controlled
Number of rules		Generated automatically based on the membership function	Total = 1st input (3) * 2nd input (3) = 9

### 3.1. Designing the first model of the fuzzy inference system FIS-1

**Fig 6:**
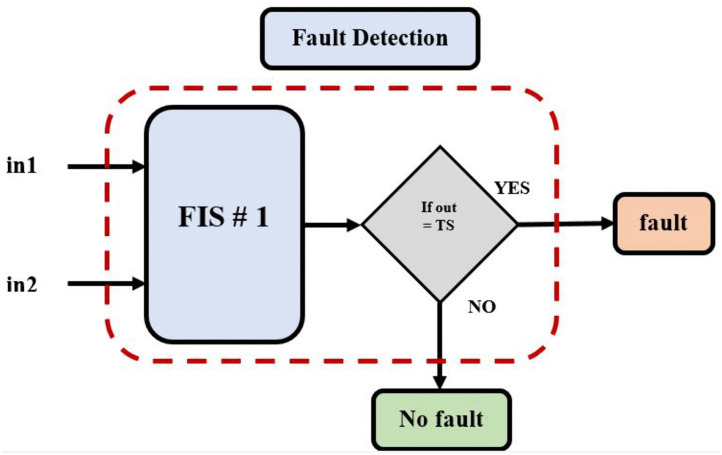
Fault detection proposed approach using FIS-1 model.

**Fig 7:**
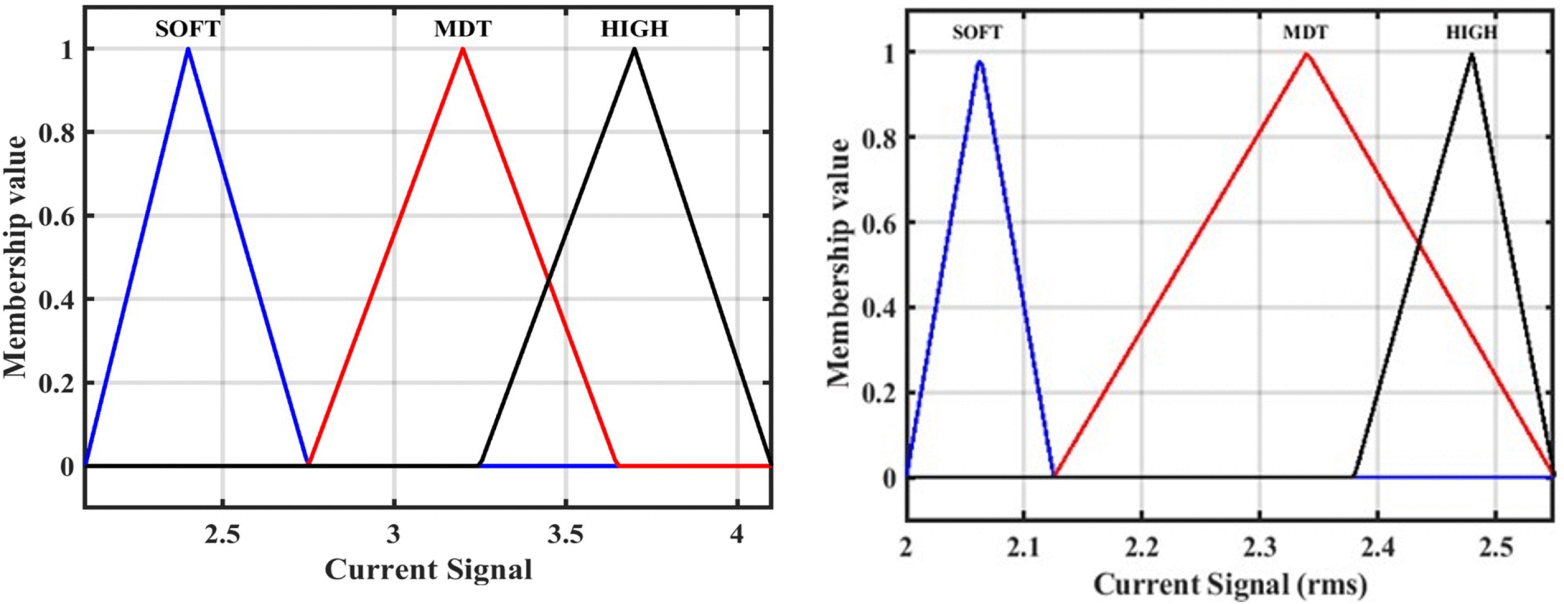
Membership function for fault detection: First input of current signal (Instantaneous current), and second input of current signal (RMS value).

As mentioned, our proposed ANFIS-based fault detection location approach contains three separate FIS models, each of which performs a specific task. In this sub-section, the first model has been described, FIS-1. It accepts a DC signal as input, detects the fault. The “Sugeno” style of fuzzy controller has been utilized to create the FIS-1. The membership function does not have any precise criteria to pick from. Triangular membership functions might be more appropriate for this application than the true and false ones discovered. To construct a Triangular membership function and signaling currents in various ranges, this membership function is designed using three bands of current as follows: a soft state (SOFT), moderate state (MDT), and high state (HIGH). The output membership function has two parts, TS (1) and FS (0), where TS stands for a trip (fault) and FS stands for non-trip (no-fault). The FIS for detecting faults is given in Fig 6. The three-membership function of fault detection has been shown in Fig 7 [33].

### 3.2. Designing the second model of the fuzzy inference system FIS-2

**Fig 8:**
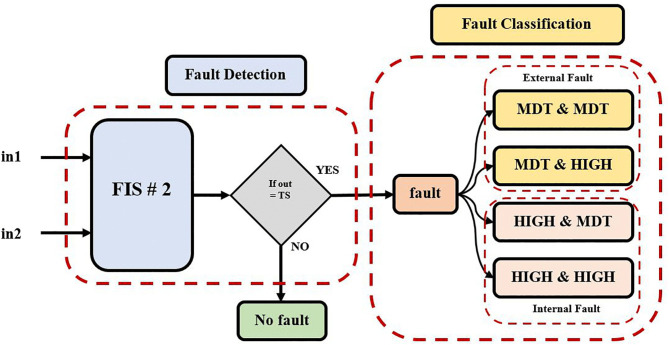
Fault classification proposed approach using FIS-2 model.

**Fig 9:**
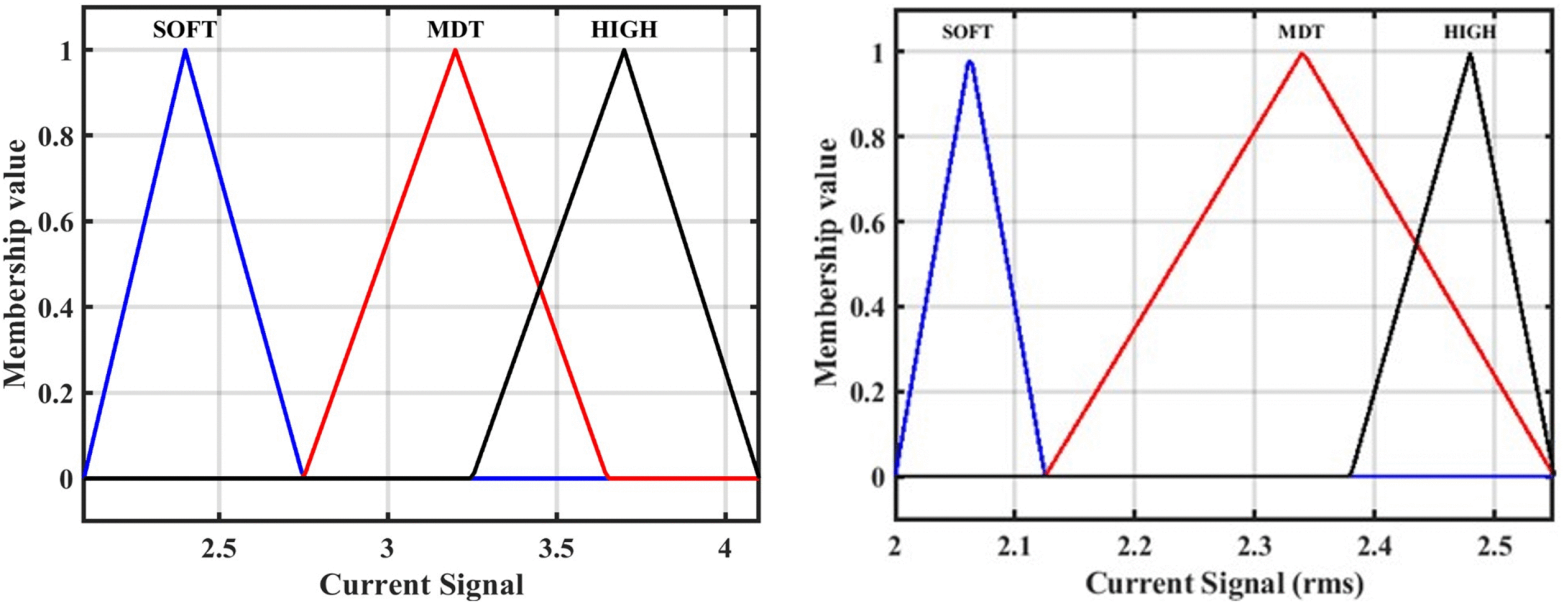
Membership function fault section identification: First input of current signal (Instantaneous current) and second input of current signal (RMS value).

The second model of FIS has been presented in this section. FIS-2 is used in the second proposed method for fault section identification [33]. Fig 8 shows the construction and operation of the proposed FIS-2 technique for fault classification. The FIS-2 module receives DC signals from the HVDC-TL as inputs. As illustrated in Fig 9, the triangle membership functions (SOFT), (MDT), and (HIGH) are used to set three bands of signal currents. The result: Internal faults are represented by TS (1), faults are represented by FS (0), while exterior faults are represented by trip TS (−1).

### 3.3. Designing the third model of the fuzzy inference system FIS-3

**Fig 10:**
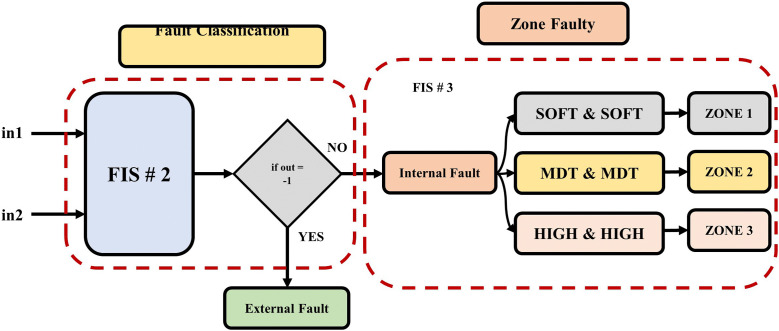
Zone faulty proposed approach utilizing FIS-3 model.

**Fig 11:**
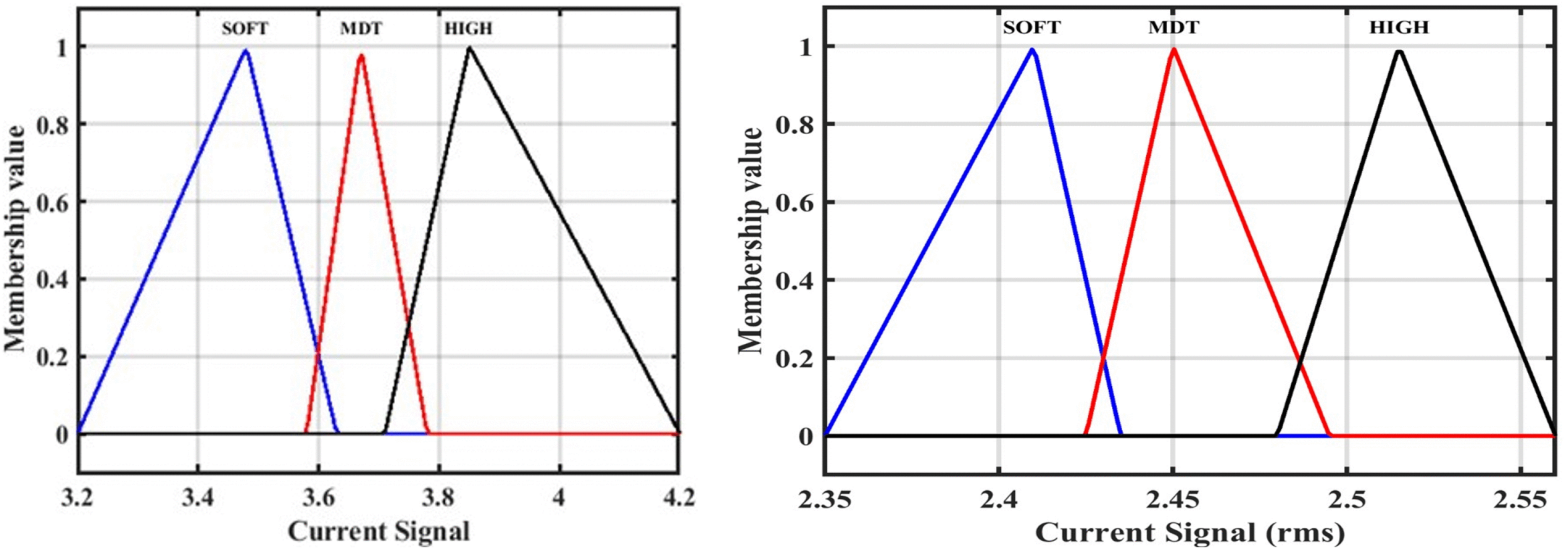
The membership function for determining the fault zone: First input of current signal (Instantaneous current), and second input of current signal (RMS value).

The third task performs the identification of the fault zone. This sub-section presents the contents and setting of the FIS-3 model in the proposed ANFIS-based fault detection/location approach. The third proposed independent method utilizes the FIS-3 fuzzy inference model. It identifies the fault zone within the DC network. Fig 10 shows the execution sequence of the FIS-3 model. The system’s input uses DC signals from the HVDC-TL. three proposed bands of input are set using triangle membership function soft state (SOFT), moderate (MDT), and high (HIGH) as shown in Fig 11. The output of the membership function is divided into three bands to determine the fault zone and boundaries of protection zones ZONE 1 from 1 km to 55 km, ZONE 2 from 45 km to 105 km, and ZONE 3 from 95 km to 150 km: ZONE 1 TS, ZONE 2 TS, and ZONE 3 TS.

## 4. Designing the ANFIS for fault location

**Fig 12:**
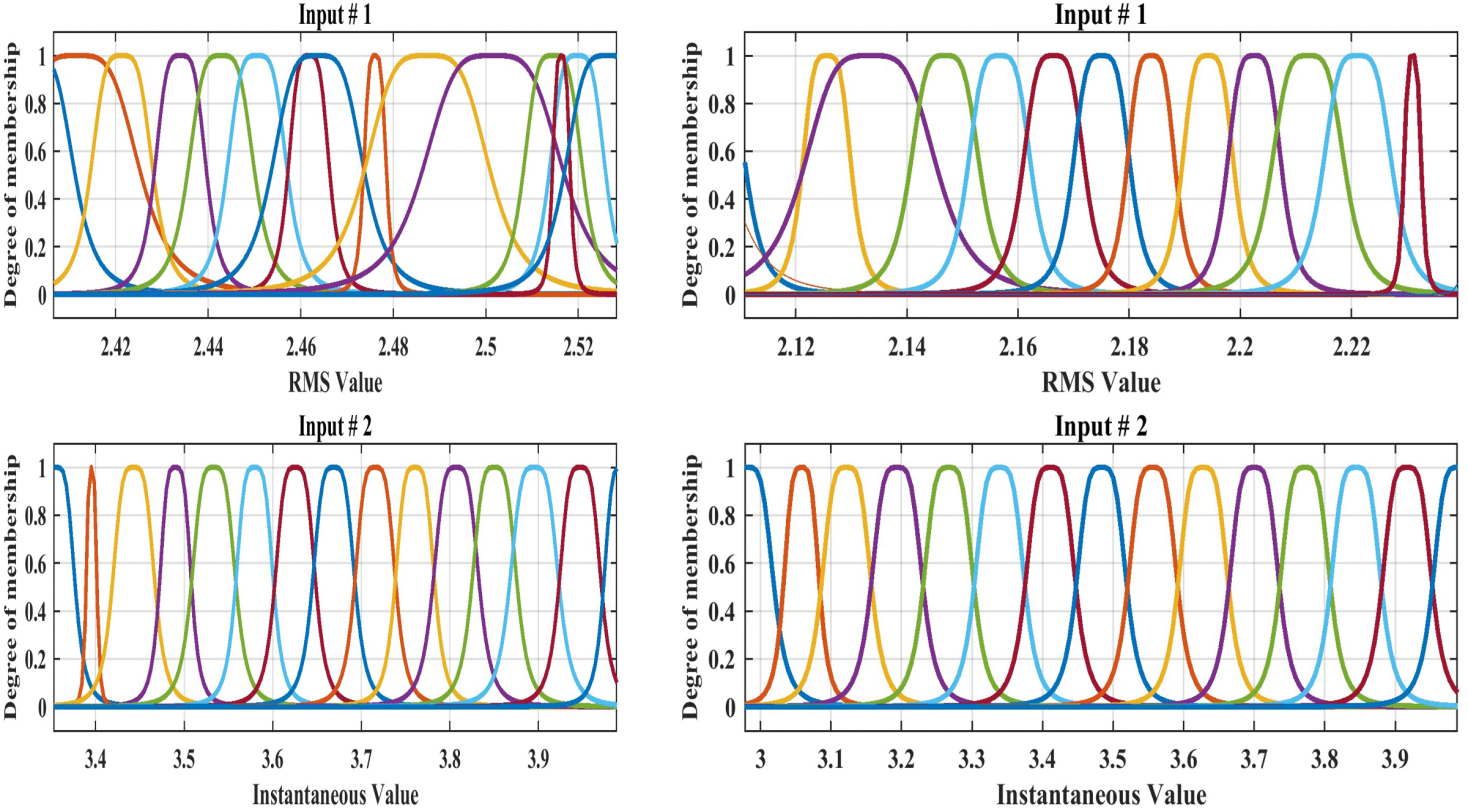
The membership function for the fault location of the ANFIS design: Conventional method, and for the correction method.

**Fig 13:**
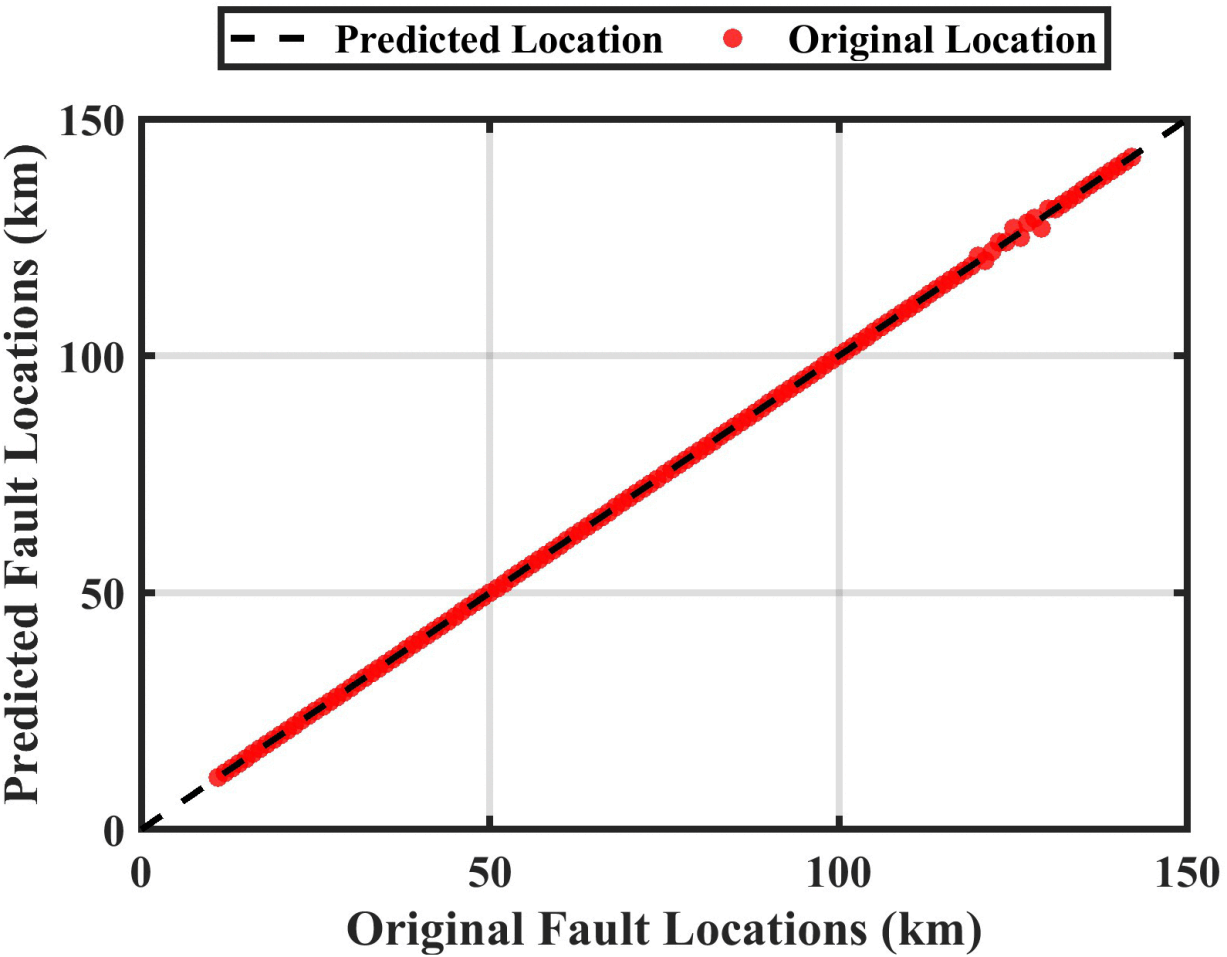
The original length vs the predicted length at zero ohms.

A system is designed to locate the fault by determining the fault kilometer through training and design of the ANFIS. The system has been studied and simulated using data of the DC signal “ DC data representing input of the ANFIS”, and the system output is the estimated fault location of the HVDC-TL. Fig 12 shows the membership function for training the ANFIS, and Fig 13 shows the predicted output with the original fault locations.

Based on the proposed designed ANFIS model, Table 4 provides a description of the ANFIS structure and training methodology. Extensive simulation experiments that were meticulously؟ crafted to simulate realistic operating circumstances and a range of failure scenarios in the system under investigation were used to validate the suggested approach. The simulation environment was constructed using realistic fault characteristics and realistic system parameters. Additional statistical analyses, including the computation of important performance metrics such as, Error %, RMSE, standard deviation, and R-squared, were carried out in order to validate the simulation results and show the effectiveness of the proposed approach in our presented research paper.

**Table 4:** Description of ANFIS Training Structure and Performance Metrics.

Item	The details of training	
	Description	Setting
**Input data**	Two inputs	Matrix [2 * 142]
**Output data**	Single output	Matrix [1 * 142]
**Type of membership function**	For input data	Generalized bell membership function ‘gbellmf’
**Number of membership functions**	Membership function/input	15 & controlled
**Number of rules**	Generated automatically based on the membership function	Total = 1^st^ input (15) * 2^nd^ input (15) = 225
**Number of Epochs**	Adjusts with minimal error	Between 50:100
**Training data**	85%	
**Testing data**	15%	
**Evaluation requirements**	Evaluation indicates (error%, RMSE, R^2^, Standard deviation)	
**Overfitting control**	Technique for preventing overfitting: error monitoring combined with limited rules	

## 5. Simulation experiments and results

The system described in the file Fig 3 was simulated. The current signals were obtained from the transmission line, and the current signal was obtained in the steady state without any faults. Several faults were executed, which are F1, F_2_, F3, and F4. Also, the faults F5 and F6 were executed. The proposed protection principle has been implemented. The fault AC/DC type, fault location, fault resistance, far-end faults, and near-end faults have all been modified. The types of faults in this research are as follows: AC faults such as single line to ground, double line, double line to ground, and triple line to ground faults. DC faults only one fault is called a single line to ground faults.

Tables 5 and 6 show the performance and results of the fuzzy inference system’s simulation in detecting the fault, determining the fault section, and determining the zone of the fault occurring under the influence of changing the fault resistance.

**Table 5:** Proposed method performance with variable fault resistance.

Case Study	Fault resistance Ω	Performance of the proposed approach (FIS 1,2)			Display	
		Reaction of FIS			Symbol	Description
—	—	0	—	—	FS	NO fault
F1	0.05	—	—	−1	TS	External fault
F2	0.05	—	1	—	TS	Internal fault
F3	0.05	—	1	—	TS	Internal fault
F4	0.05	—	1	—	TS	Internal fault
F3	100	—	1	—	TS	Internal fault
F4	100	—	1	—	TS	Internal fault
F1	300	—	—	−1	TS	External fault
F2	300	—	1	—	TS	Internal fault
F5	100	—	1	—	TS	Internal fault
F6	300	—	—	−1	TS	External fault

**Table 6:** Determining the fault zone with variable fault resistance.

Case study NO.	Fault location km	Fault resistance Ω	Performance of the proposed approach (FIS-3)		
			Reaction of FIS 3 (Q3)		
1	15	0.05	0.5003	TS	ZONE 1
2	50		1.00	TS	ZONE 1
3	80		1.5437	TS	ZONE 2
4	110		2.4998	TS	ZONE 3
5	140		2.5	TS	ZONE 3
6	15	100	0.4	TS	ZONE 1
7	50		0.8378	TS	ZONE 1
8	80		1.5	TS	ZONE 2
9	110		2.490	TS	ZONE 3
10	140		2.5	TS	ZONE 3
11	15	300	0.5015	TS	ZONE 1
12	50		0.8464	TS	ZONE 1
13	80		1.5000	TS	ZONE 2
14	110		2.4998	TS	ZONE 3
15	140		2.490	TS	ZONE 3

### 5.1. Performance evaluation of ANFIS for fault location

To restore the functionality of the entire HVDC system as rapidly as feasible, an accurate assessment of the fault site is critical. To test the accuracy of the suggested approach for locating faults with various fault resistance values, 142 DC fault circumstances were utilized to determine the highest instantaneous current value and the rms value [34]. The models used in this study were evaluated using four distinct performance metrics in order to give a thorough evaluation of the accuracy and effectiveness of the forecasted outcomes. Beyond percentage error, we have now integrated widely accepted statistical markers to provide a more comprehensive evaluation. Specifically, the coefficient of determination (R^2^) is employed to assess the goodness of fit between predicted and actual values. At the same time, the root mean square error (RMSE) is used to quantify the overall variance and prediction accuracy. Additionally, the standard deviation (SD) is used to capture the variability of results, complementing the error percentage. The relative error % for each fault instance was determined using equation (1), where *L*_*orig*_ is the original length from the rectifier substation to the fault point, *L*_*pred*_ is the proposed method’s assumed fault location, and *L*_*Total*_ is the total length of the DC transmission line.


$$E(\%)=\left|\frac{L_{orig}-L_{pred}}{L_{Total}}\right|*\text{1}\text{0}\text{0}$$ (1)

Taking the square root of the MSE, as shown in Equation (2), yields the root mean squared error (RMSE) [35].


$$RMSE=\;\sqrt{\frac{\text{1}}{n}\sum_{i=\text{1}}^{n}(L_{orig}-\;L_{pred}})²$$ (2)

In power transmission systems, the R-squared (R^2^) statistic is frequently used to assess the performance of fault location algorithms, as shown in Equation (3).


$$R^{\text{2}}=\text{1}-{\left(\frac{\left(L_{orig}-\;L_{pred}\right)}{L_{orig}}\right)}^{\text{2}}$$ (3)

Lastly, use Equation (4) to determine the standard deviation between each pair of data.


$$SD=\;\sqrt{\frac{{\left(L_{orig}-\;L_{mean}\right)}^{\text{2}}+{\left(L_{pred}-L_{mean}\right)}^{\text{2}}}{\text{2}}}$$ (4)

**Fig 14:**
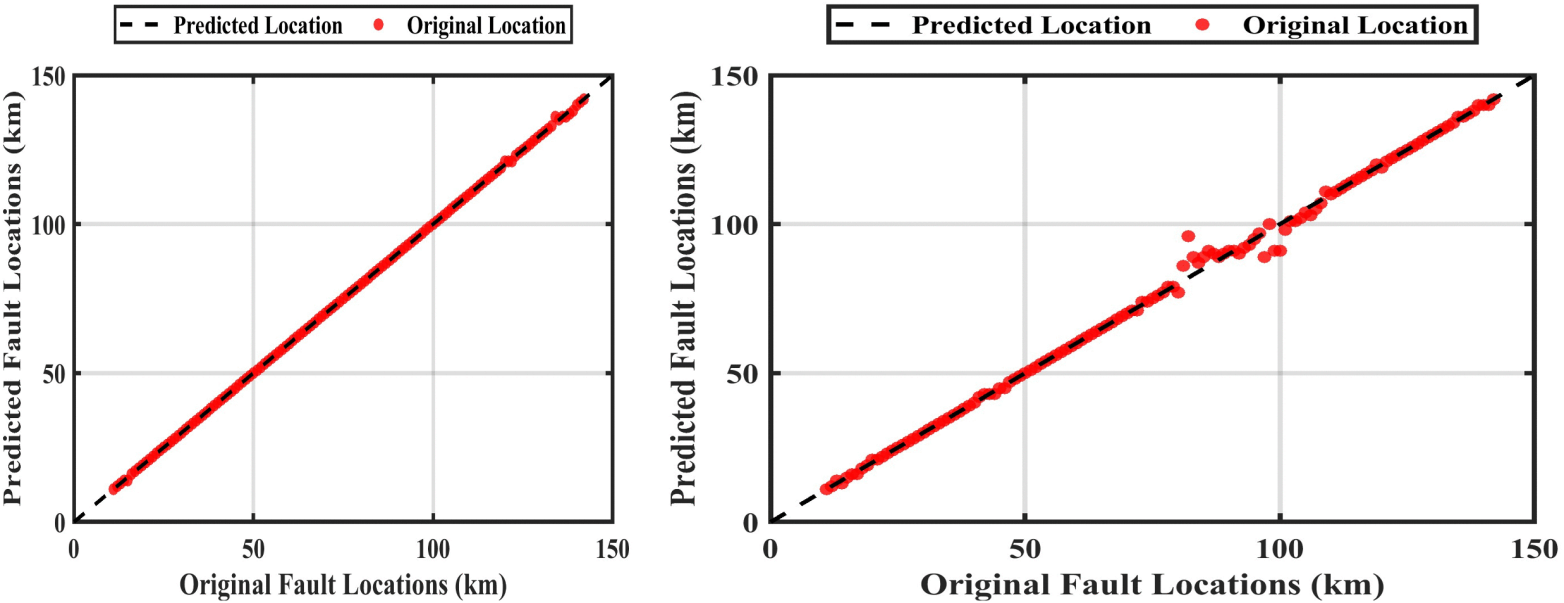
The original length vs the expected length “conventional method” at fault resistances of 0.05 Ω and 300 Ω: With fault resistance 0.05 Ω and at fault resistance 300 Ω.

Where *L*_*mean*_ the mean between two values original and predicted. Fig 14 shows the training of the ANFIS to locate the fault at the fault resistance of 0.05 Ω and 300 Ω. Table 7 shows the determination of the fault location with the selection of the maximum instantaneous current value and the maximum rms value. This method is called the conventional method.

**Table 7:** Fault location with the variable resistance (conventional method).

Case study No.	Original fault location (km)	Fault resistance Ω	Fault Location Technique Performance				
			Predicted fault location (km)	Error percentage (%)	RMSE	R-squared	Standard deviation
1	15	0.05	14.4910	0.3393	0.5090	0.9988	0.2545
2	50		49.9752	0.0165	0.0248	1.0000	0.0124
3	80		80.0395	0.0263	0.0395	1.0000	0.0198
4	110		110.0526	0.0351	0.0526	1.0000	0.0263
5	140		140.3426	0.2284	0.3426	1.0000	0.1713
6	15	100	14.7654	0.1564	0.2346	0.9998	0.1173
7	50		49.8488	0.1008	0.1512	1.0000	0.0756
8	80		80.1615	0.1077	0.1615	1.0000	0.0808
9	110		110.3932	0.2621	0.3932	1.0000	0.1966
10	140		141.2394	0.8263	1.2394	0.9999	0.6197
11	15	300	15.0724	0.0483	0.0724	1.0000	0.0362
12	50		50.0289	0.0193	0.0289	1.0000	0.0145
13	80		76.9158	2.0561	3.0842	0.9985	1.5421
14	110		110.4679	0.3119	0.4679	1.0000	0.2340
15	140		140.0047	0.0031	0.0047	1.0000	0.0024

**Fig 15:**
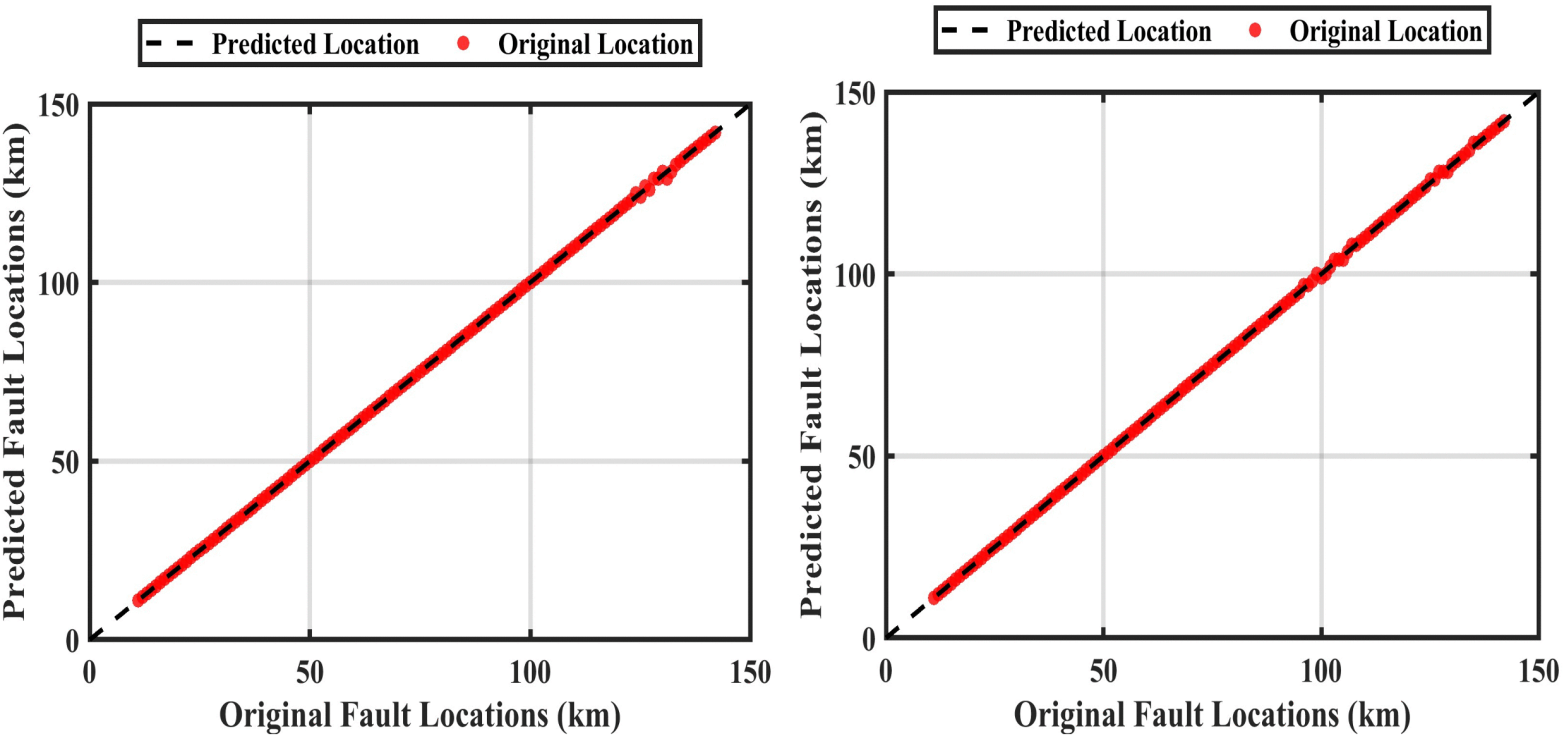
Comparison between the original and predicted fault locations using the correction method at fault resistances of 0.05 Ω and 300 Ω: For 0.05 Ω and 300 Ω.

A proposed method was found to deal with this problem by obtaining the maximum value of the instantaneous current and the maximum effective value at a fixed time value during the failure period, the fault application time is 0.3 sec, the fault persistence time is 0.2sec, and during this period the time was fixed at 0.3015sec, this method is called the ‘Correction Method’ Fig 15 shows the training of the ANFIS at a constant time with an fault resistance of 0.05 Ω and 300 Ω. Table 8 shows the fault location with a constant time of 0.3015 sec.

**Table 8:** Fault location with the variable resistance (correction method).

Case study No.	Original fault location (km)	Fault resistance Ω	Fault Location Technique Performance				
			Predicted fault location (km)	Error percentage (%)	RMSE	R-squared	Standard deviation
1	15	0.05	14.8968	0.0688	0.1032	1.0000	0.0516
2	50		50.0063	0.0042	0.0063	1.0000	0.0032
3	80		79.9248	0.0501	0.0752	1.0000	0.0376
4	110		109.9768	0.0155	0.0232	1.0000	0.0116
5	140		140.0725	0.0483	0.0725	1.0000	0.0362
6	15	100	14.9714	0.0191	0.0286	1.0000	0.0143
7	50		49.9169	0.0554	0.0831	1.0000	0.0416
8	80		80.0173	0.0115	0.0173	1.0000	0.0087
9	110		108.7955	0.8030	1.2045	0.9999	0.6022
10	140		140.1106	0.0737	0.1106	1.0000	0.0553
11	15	300	15.0286	0.0191	0.0286	1.0000	0.0143
12	50		49.9927	0.0049	0.0073	1.0000	0.0037
13	80		79.9660	0.0227	0.0340	1.0000	0.0170
14	110		109.8939	0.0707	0.1061	1.0000	0.0530
15	140		140.0370	0.0247	0.0370	1.0000	0.0185

**Fig 16:**
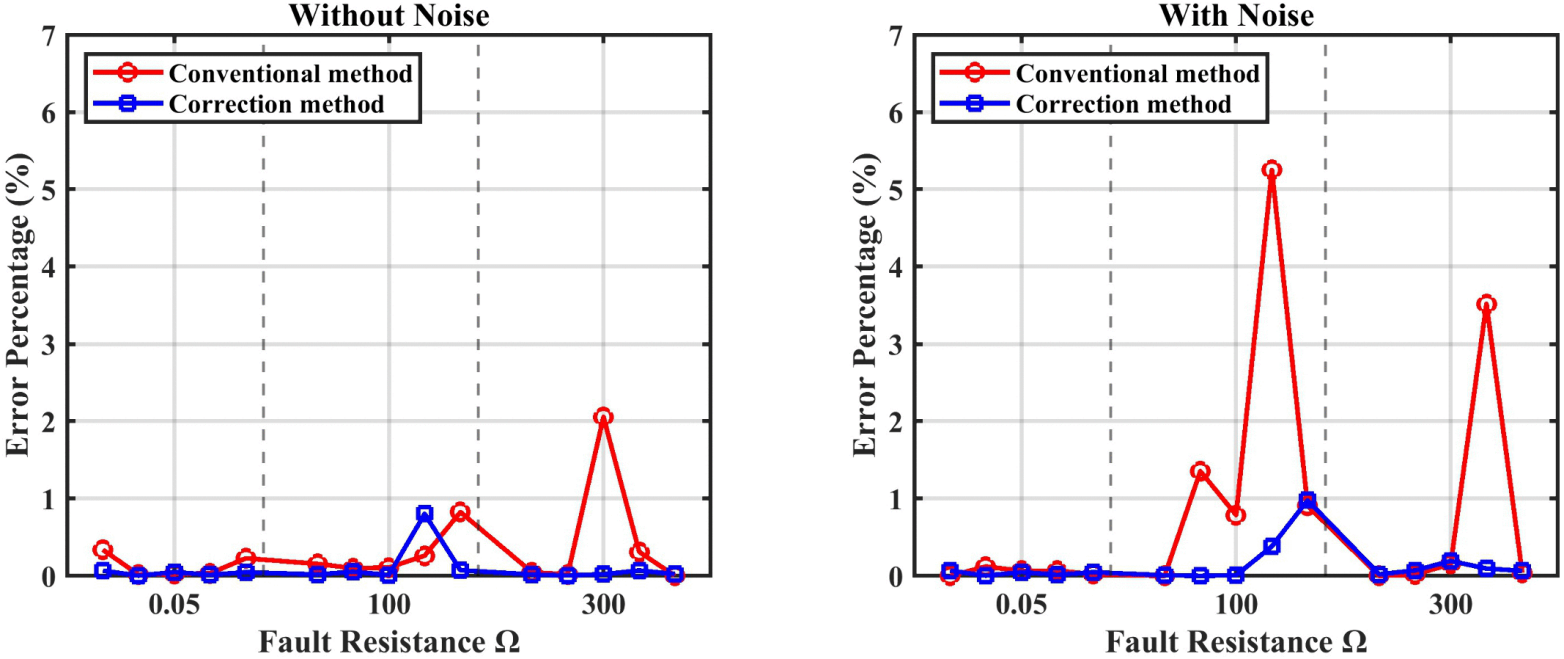
The comparison between the conventional method and the correction method.

**Fig 17:**
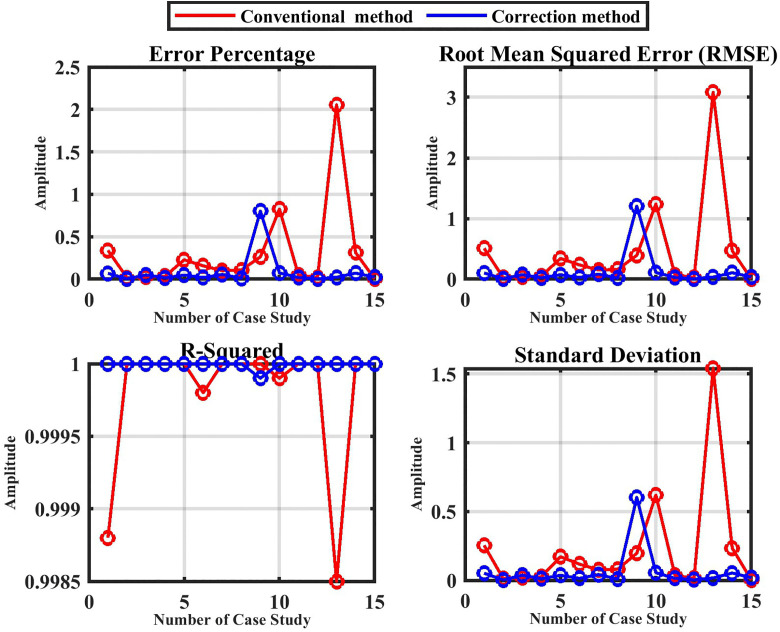
Statistical analysis for the proposed technique without noise.

Fig 16 also shows the error percentage with various fault resistance (0.05, 100, and 300) Ω between the two methods (conventional and corrected). The statistical analysis presented in Tables 7 and 8 for both the conventional method and the corrected method is displayed in Fig 17.

We have elaborated on the data collection process. Specifically, 142 RMS value cases and 142 instantaneous current value cases were obtained, one case per kilometer along the 150 km transmission line, resulting in a total of 284 simulation cases. This ensures that the dataset is both systematic and statistically meaningful.

**Fig 18:**
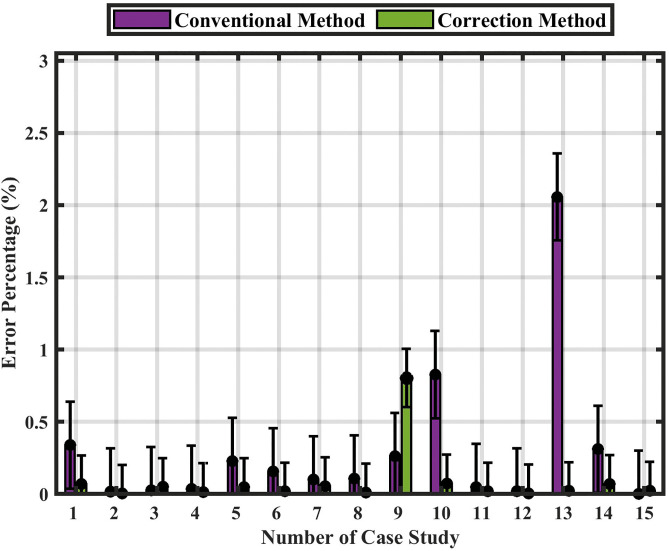
Statistical comparison of the conventional and correction methods using error-bar analysis without noise.

Fig 18 Performance comparison with error bars representing standard deviation across 142 simulation cases. The inclusion of error bars illustrates the variability and statistical confidence of the results, enhancing the robustness and transparency of the performance evaluation.

### 5.3. Noise interference effect

**Fig 19:**
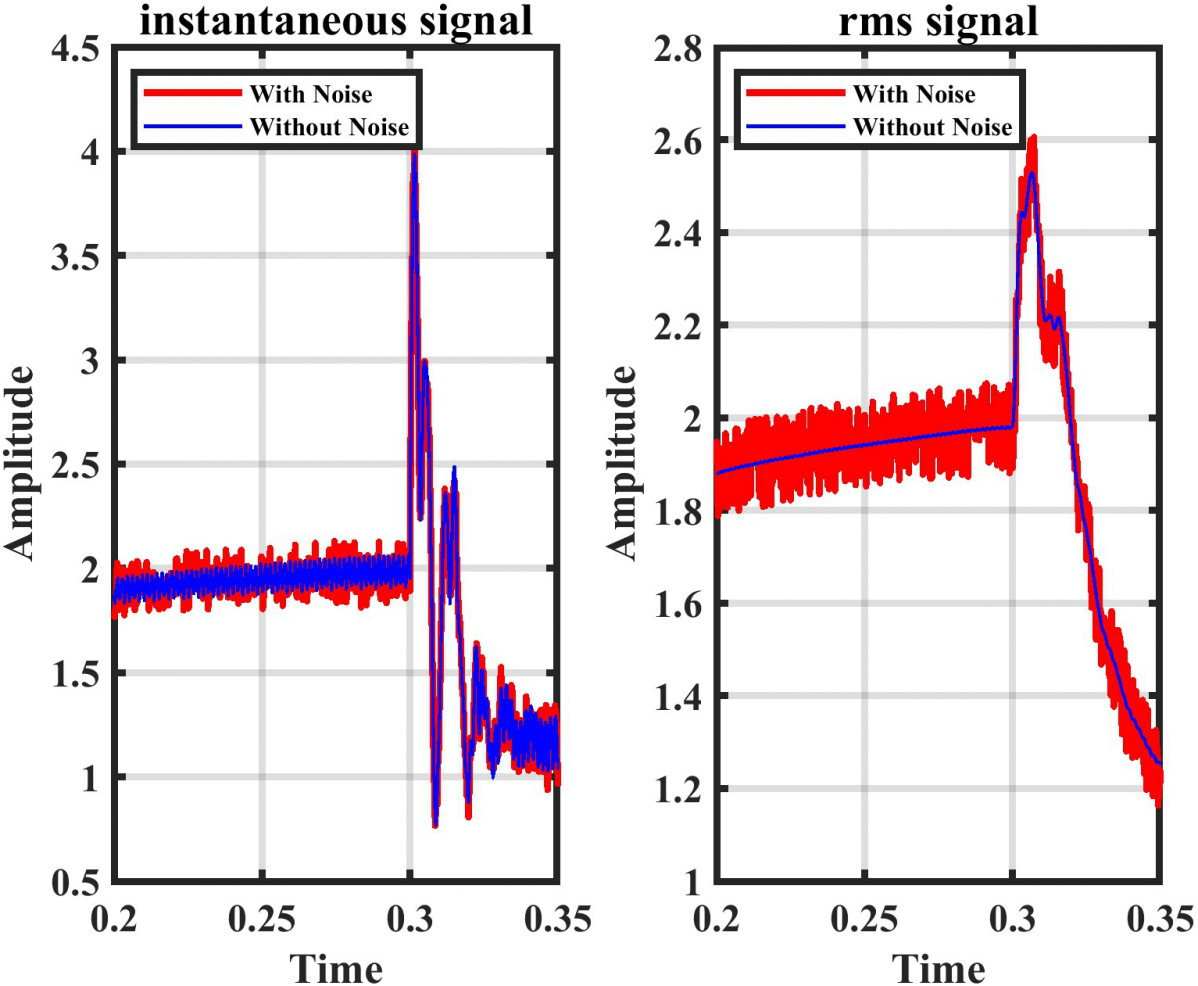
The current signal under noise interference effect.

HVDC-TLs are exposed to some sources of noise, and this affects the determination of the location of the fault. and this noise affects the value of the current, whether it increases or decreases, and also the shape of the current wave. Therefore, the proposed method must have the ability and efficiency to deal with noise that affects the system and locate the fault with high accuracy. The noise level that affected the system was 26 dB SNR, as shown in Fig 19. The findings compiled in Tables 9 and 10 demonstrate the effectiveness of the proposed technique in identifying faults under noise conditions.

**Table 10:** The performance of the proposed methodology for fault location (Correction Method) under the noise effect.

Case study No.	Original Fault location (km)	Fault resistance Ω	SNR (dB)	Fault location technique performance				
				Predicted fault location (km)	Error percentage (%)	RMSE	R-squared	Standard deviation
1	15	0.05	26	14.8968	0.0688	0.1032	1.0000	0.0516
2	50		26	50.0063	0.0042	0.0063	1.0000	0.0032
3	80		26	79.9248	0.0501	0.0752	1.0000	0.0376
4	110		26	109.9768	0.0155	0.0232	1.0000	0.0116
5	140		26	140.0725	0.0483	0.0725	1.0000	0.0362
6	15	100	26	14.9874	0.0084	0.0126	1.0000	0.0063
7	50		26	50.0029	0.0019	0.0029	1.0000	0.0014
8	80		26	79.9863	0.0091	0.0137	1.0000	0.0069
9	110		26	110.5748	0.3832	0.5748	1.0000	0.2874
10	140		26	138.5319	0.9787	1.4681	0.9999	0.7340
11	15	300	26	15.0321	0.0214	0.0321	1.0000	0.0160
12	50		26	49.8940	0.0707	0.1060	1.0000	0.0530
13	80		26	79.7086	0.1943	0.2914	1.0000	0.1457
14	110		26	110.1443	0.0962	0.1443	1.0000	0.0722
15	140		26	139.9015	0.0657	0.0985	1.0000	0.0493

**Table 9:** The performance of the proposed methodology for fault location (Conventional Method) under the noise effect.

Case study No.	Original fault location (km)	Fault resistance Ω	SNR (dB)	Fault Location Technique Performance				
				Predicted fault location (km)	Error percentage (%)	RMSE	R-squared	Standard deviation
1	15	0.05	26	15.0007	0.0005	0.0007	1.0000	0.0004
2	50		26	49.8166	0.1223	0.1834	1.0000	0.0917
3	80		26	79.9011	0.0659	0.0989	1.0000	0.0495
4	110		26	109.8977	0.0682	0.1023	1.0000	0.0511
5	140		26	140.0336	0.0224	0.0336	1.0000	0.0168
6	15	100	26	15.0001	0.0001	0.0001	1.0000	0.0000
7	50		26	47.9675	1.3550	2.0325	0.9983	1.0162
8	80		26	78.8182	0.7879	1.1818	0.9998	0.5909
9	110		26	117.8727	5.2485	7.8727	0.9949	3.9363
10	140		26	138.6374	0.9084	1.3626	0.9999	0.6813
11	15	300	26	14.9997	0.0002	0.0003	1.0000	0.0001
12	50		26	50.0264	0.0176	0.0264	1.0000	0.0132
13	80		26	79.7698	0.1535	0.2302	1.0000	0.1151
14	110		26	115.2771	3.5181	5.2771	0.9977	2.6386
15	140		26	140.0628	0.0419	0.0628	1.0000	0.0314

The performance of the proposed method at different noise levels (20, 25, and 30 dB) is summarized in Table 11, confirming the effectiveness and resilience of the proposed approach in accurately identifying and locating faults under noisy operating conditions.

**Table 11:** The performance of the proposed methodology for fault location (Conventional Method &Correction Method) under different noise levels.

Case Study No.	Method	Original fault location (km)	Fault resistance Ω	SNR (dB)	Fault location technique performance				
					Predicted fault location (km)	Error percentage (%)	RMSE	R-squared	Standard deviation
1	Conventional	15	0.05	20	15.0001	0.0001	0.0001	1.0000	0.0000
2		50	0.05	20	49.5473	0.3018	0.4527	0.9999	0.2264
3		80	100	25	74.7775	3.4817	5.2225	0.9957	2.6112
4		100	100	25	104.8072	3.2048	4.8072	0.9977	2.4036
5		110	300	30	110.0005	0.0003	0.0005	1.0000	0.0003
6		140	300	30	135.5457	2.9695	4.4543	0.9990	2.2271
7	Correction	15	0.05	20	14.9996	0.0003	0.0004	1.0000	0.0002
8		50	0.05	20	49.9994	0.0004	0.0006	1.0000	0.0003
9		80	100	25	80.0173	0.0115	0.0173	1.0000	0.0087
10		100	100	25	99.4629	0.3581	0.5371	1.0000	0.2685
11		110	300	30	110.0024	0.0016	0.0024	1.0000	0.0012
12		140	300	30	139.9998	0.0001	0.0002	1.0000	0.0001

**Fig 20:**
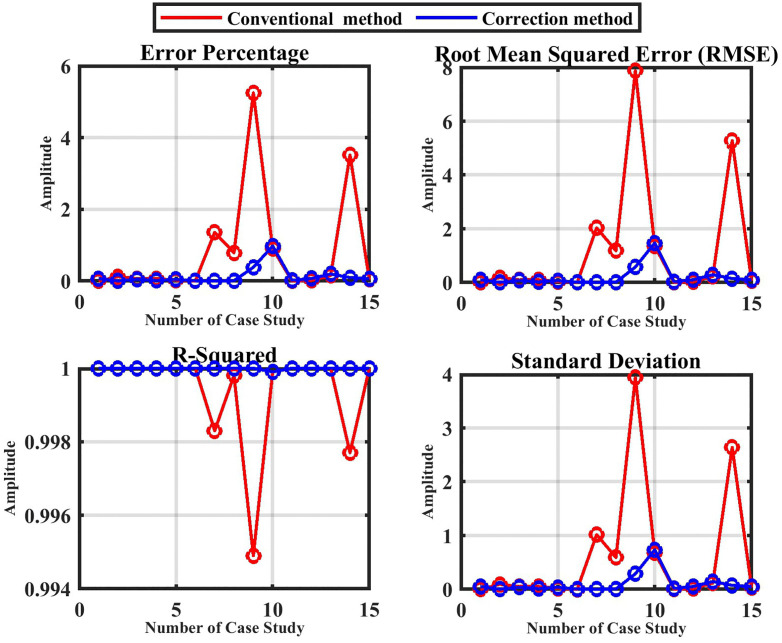
Statistical analysis for the proposed technique with noise.

In contrast to the previous results without noise (Fig 17), Fig 20 presents the statistical analysis of both the conventional and enhanced methods under the effect of noise, as summarized in Tables 9 and 10.

**Fig 21:**
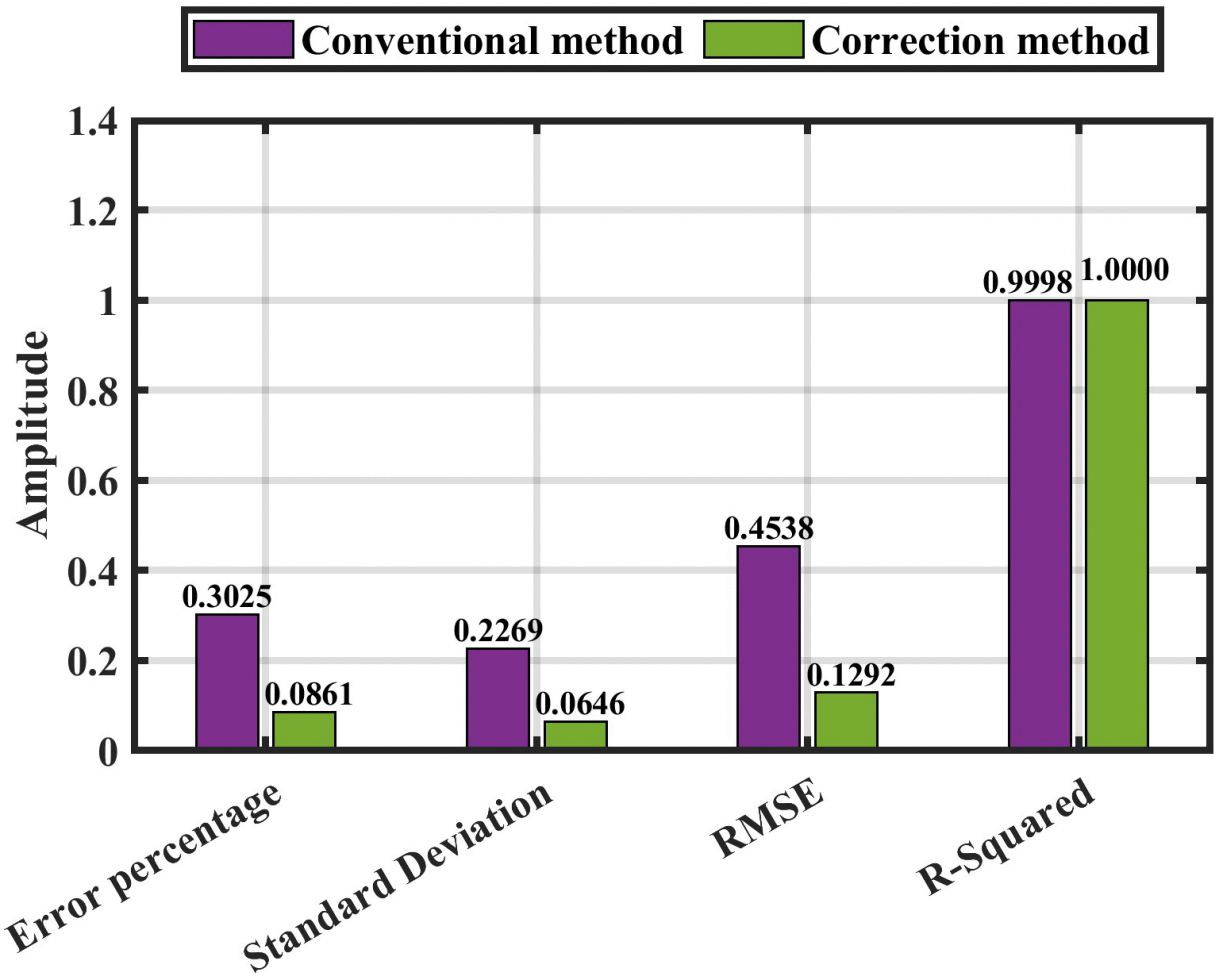
The overall evaluation of the proposed method without noise.

**Fig 22:**
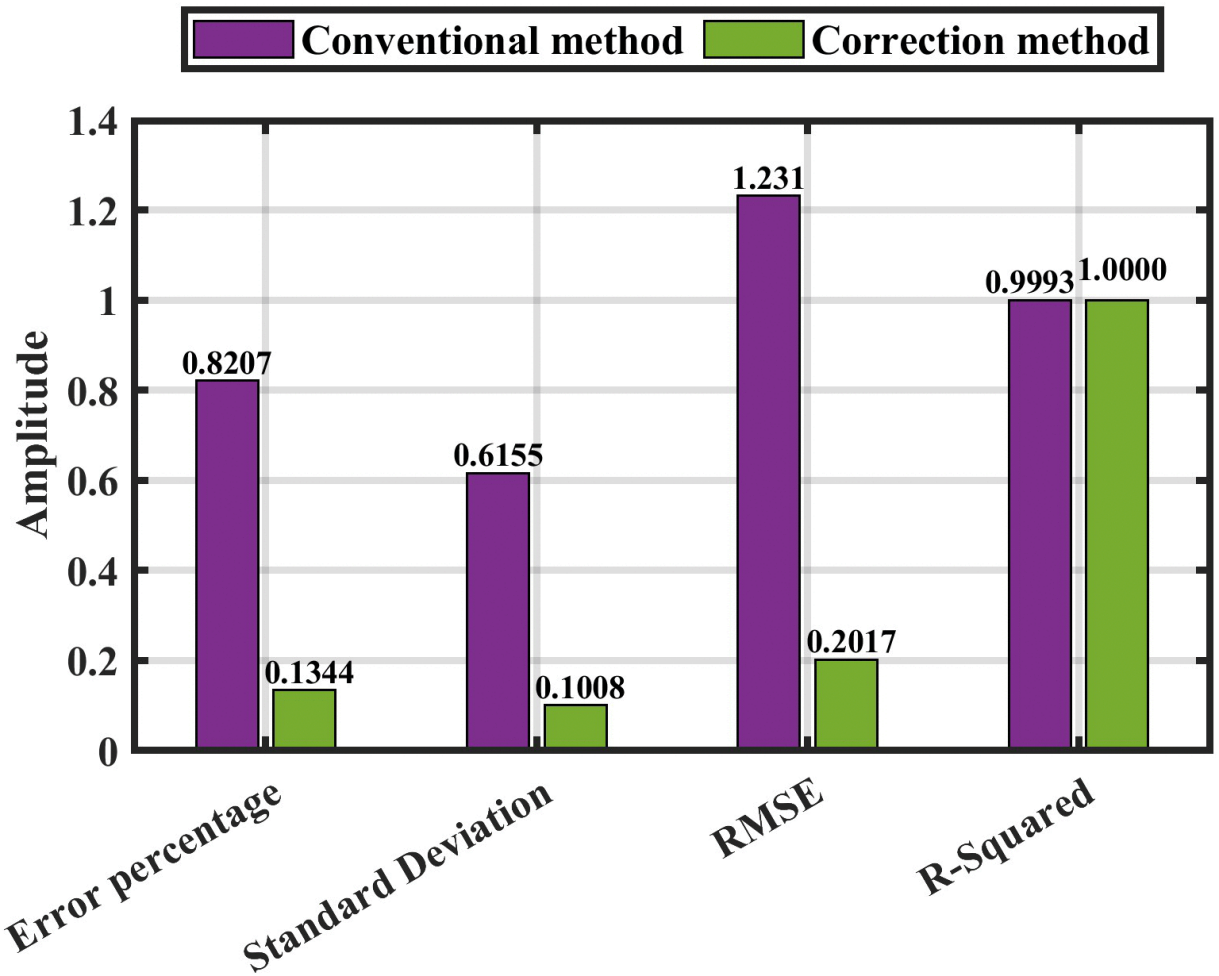
The overall evaluation of the proposed method with noise.

Finally, the overall evaluation of the suggested approach, summarized in Table 12 and illustrated in Figs 21 and 22, highlight the method’s capability to achieve stable and precise performance across a wide range of operating conditions, confirming its robustness and effectiveness in HVDC fault detection and localization.

**Table 12:** Evaluation of the proposed method.

Aspect	Without Noise		With Noise	
	Conventional Method	Correction Method	Conventional Method	Correction Method
Error Percentage (%)	0.3025	0.0861	0.8207	0.1344
RMSE	0.4538	0.1292	1.2310	0.2017
R-Squared	0.9998	1.0000	0.9994	1.0000
Standard Deviation	0.2269	0.0646	0.6155	0.1008

**Fig 23:**
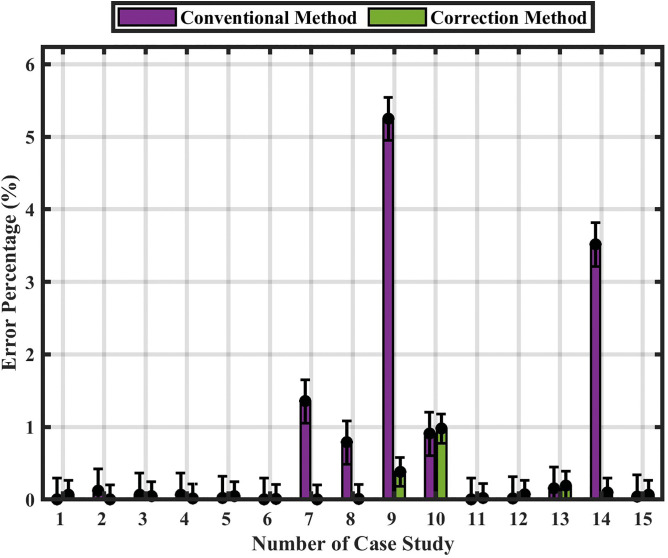
Statistical comparison of the conventional and correction methods using error-bar analysis with noise.

The inclusion of error bars in Fig 23 clearly illustrates the variability of the results, demonstrating the enhanced stability and robustness of the correction method compared with the conventional one under noisy conditions.

### 5.4. Comparison with different protection systems

The techniques used to identify, classify, and locate faults in HVDC-TLs are briefly compared in this section. The fault with a fault resistance of 100 ohms and an error rate of 1% is detected and located using an algorithm [9]. The gap frequency spectrum, and the fault with a fault resistance of 100 ohms and an error rate of 0.640% is located using the algorithm [36]. Natural frequency. [37,38] and [39] are used to detect, classify, and locate faults; [37] has an error rate of 0.2% when the fault resistance is 300 ohms, and under the influence of 30 dB noise. [38] and [39] have an error of 0.658% when the fault resistance is 100 ohms, and [39] has a large error of up to 29%. [40] is predicated on identifying and classifying faults with noise levels of 20–30 dB and fault resistances of up to 100 ohms. Both [41] and [42] rely on locating faults [41] with fault resistances up to 50 ohms that have not been investigated under the influence of noise (error rate: 1.2%), while [42] investigates fault locations with fault resistances up to 100 ohms that have not been examined under the influence of noise (error rate: 5%). The performance of the suggested strategy with various algorithms is displayed in Table 13, which also presents the method’s outcomes from this study.

**Table 13:** The proposed method’s performance compared to some of the proposed protection methods.

Paper. No	The approach made	Fault detection	Fault classification	Fault location	Fault resistance (Ω)	Noise level (dB)	Max.error without noise (%)	Max.error with noise (%)
[9]	Gap frequency spectrum	yes	Not mentioned	yes	100	Not mentioned	1%	—
[36]	Natural frequency	Not mentioned	Not mentioned	yes	100	Not mentioned	0.640	—
[37]	Transient current	yes	yes	yes	300	30	0.2	1.68
[38]	ANFIS + DWT	yes	yes	yes	100	Not mentioned	0.658	—
[39]	MODWT	yes	yes	yes	100	yes	29	—
[40]	Fault loop analysis	yes	yes	no	100	20	—	—
						30		
[41]	Time-domain transient-based method	Not mentioned	Not mentioned	yes	100	Not mentioned	1.2	—
[42]	Simple single-ended, post-fault technique	Not mentioned	Not mentioned	yes	50	Not mentioned	5	—
This work	FIS+ANFIS	yes	yes	yes	300	20	0.0861	0.3581
						25		
						26		
						30		

## 6. Conclusion

This study presents a novel protection mechanism for HVDC transmission lines that integrates FIS for fault detection, classification, and zone identification with an ANFIS for precise fault location. The proposed scheme demonstrates high accuracy in locating faults under diverse operating conditions, including variable fault resistances up to 300 Ω and noisy environments, where the correction method further reduced error percentages compared to conventional techniques. The results confirm that the approach enhances fault detection speed and reliability, offering a practical alternative to more complex existing methods.

### 6.1. Further points to be investigated

While the present study demonstrates promising results, some limitations should be acknowledged. The evaluation is based on PSCAD and MATLAB simulations, which, although comprehensive, may not fully reflect the complexity of real-world HVDC networks. In addition, the approach could be influenced by parameter variations, such as noise levels and system settings, and certain modelling assumptions may affect its direct applicability to practical systems. Future work will therefore focus on real-time validation, hardware-in-the-loop testing, and integration with SCADA-based monitoring systems, alongside extending the approach to multi-terminal HVDC and hybrid AC/DC networks to ensure scalability and practical deployment.

## Supporting information

S1 File All image datasets were obtained from standard MATLAB image libraries (MathWorks), except for one self-captured image (Fig 1). The supporting numerical data used to generate the Irms and Iins plots are provided in the supporting information file (S1_supporting information.xlsx). https://docs.google.com/spreadsheets/d/1MlkQYagaK0DQy4E4BvPjWvEwAEFepXnR/edit?usp=sharing&ouid=103627301778289813628&rtpof=true&sd=true.
(XLSX)
